# Feasibility of Polylactic Acid–Polycaprolactone-Blended Microspheres in Transarterial Embolization Therapy

**DOI:** 10.1021/acsomega.6c00948

**Published:** 2026-07-01

**Authors:** Yi-Xin Liu, Susaritha Ramanathan, Chen-Ying Su, Li-Han Chen, Yu-Chien Lin, Ren-Jei Chung, Hsu-Wei Fang

**Affiliations:** † Department of Chemical Engineering and Biotechnology, 34877 National Taipei University of Technology, Taipei 10608, Taiwan; ‡ High-value Biomaterials Research and Commercialization Center, National Taipei University of Technology (Taipei Tech), Taipei 10608, Taiwan; § Institute of Oral Tissue Engineering and Biomaterials, National Yang Ming Chiao Tung University, Taipei 112, Taiwan; ∥ School of Materials Science and Engineering, 54761 Nanyang Technological University, 50 Nanyang Avenue, Singapore 639789, Singapore; ⊥ BIOBOND LTD, Corsham, Wiltshire SN13 0B, U.K.; # Institute of Biomedical Engineering and Nanomedicine, National Health Research Institutes, No. 35, Keyan Road, Zhunan Town, Miaoli 35053, Taiwan

## Abstract

Transarterial embolization (TAE) is a minimally invasive endovascular therapy used for tumor devascularization, hemorrhage control, and the treatment of vascular malformations. In particulate TAE, embolic microspheres are delivered through microcatheters to occlude target vessels, and the clinical outcome strongly depends on the particle uniformity, deliverability, mechanical compliance, and intravascular biocompatibility. However, currently available biodegradable embolic materials often degrade too rapidly, leading to premature recanalization, and their acidic degradation products may induce local inflammatory responses. Therefore, degradable microspheres with tunable mechanical properties and a controllable degradation behavior are needed. In this study, polylactic acid/polycaprolactone (PLA/PCL) blend microspheres were fabricated using an emulsion solvent evaporation method with varying PLA/PCL ratios and systematically evaluated. Incorporation of PCL modulated microsphere surface morphology and improved particle deformability, while compression testing demonstrated a progressive reduction in apparent Young’s modulus compared with neat PLA. Hemolysis and cytotoxicity assays confirmed that the P­(LA/CL)­73 formulation exhibited nonhemolytic behavior and good cytocompatibility. In addition, P­(LA/CL)­73 demonstrated enhanced thrombus formation, controlled and sustained degradation, and improved distal embolization performance in a 3D-printed in vitro vascular model. These findings suggest that P­(LA/CL) blend microspheres, particularly P­(LA/CL)­73, represent a promising biodegradable embolic platform with tunable mechanical and degradation properties for next-generation TAE applications.

## Graphical abstract

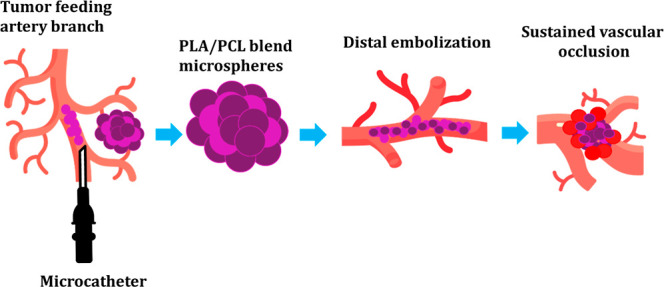

## 1 Introduction

Transarterial embolization (TAE) and related endovascular procedures have become cornerstone minimally invasive therapies for a wide range of diseases, including tumor control, hemorrhage management, and treatment of vascular malformations.^1,2^ By navigating catheters under real-time imaging from superficial access vessels to distal targets, clinicians can selectively occlude pathological blood supply with reduced surgical trauma and faster recovery compared with open surgery.^3–5^ Embolic agents are biocompatible materials delivered intravascularly via a catheter to intentionally occlude targeted vessels, producing temporary or permanent cessation of flow depending on therapeutic intent.^6,7^ Embolic agents are available in multiple physical forms, including liquid formulations, foams, particulates, and discrete particles. Among these, microparticulate embolics, especially microspheres, which are three-dimensional (3D) spherical particles on the micrometer scale, are the most widely used in clinical practice.^8^ In particulate embolization, microspheres are delivered through microcatheters to obstruct flow at predefined vascular levels, enabling localized ischemia or hemostasis.^3,9,10^ Clinical success, therefore, depends strongly on deliverability, size and shape uniformity, mechanical compliance, and biological response of the embolic agents within the vessel.^11,12^

Commercial embolic materials span nondegradable and degradable classes.^8^ Nondegradable agents (e.g., calibrated poly­(vinyl alcohol) or acrylic microspheres) provide durable occlusion but may remain permanently within vessels, potentially contributing to chronic inflammation, loss of vascular compliance, and long-term complications.^13^ In contrast, biodegradable embolics such as gelatin sponge, degradable starch microspheres (DSMs), and poly­(lactic-*co*-glycolic acid) (PLGA)-based particles aim to restore perfusion after a therapeutic window.^14–16^ However, existing degradable agents frequently suffer from clinically relevant limitations: (i) overly rapid degradation that risks incomplete or short-lived embolization, (ii) unpredictable recanalization profiles, and (iii) inflammatory reactions triggered by acidic degradation products, which can lead to tissue irritation and postoperative discomfort.^17–20^ These constraints highlight the need for next-generation biodegradable microspheres with tunable mechanical and degradation behavior while maintaining favorable hemocompatibility and biocompatibility.

Polylactic acid (PLA) is an attractive biocompatible and biodegradable polymer due to its established safety and widespread use in resorbable implants and drug delivery.^21,22^ Biodegradable synthetic polymers have attracted considerable attention in biomedical engineering because their mechanical properties, degradation behavior, and biological interactions can be tailored through molecular design and material processing strategies.^23–25^ These polymers have been widely explored for diverse biomedical applications, including drug delivery systems, implantable devices, and advanced therapeutic materials.^23–25^ However, PLA is intrinsically brittle and comparatively stiff, and its hydrolysis generates lactic acid, which may lower local pH and exacerbate inflammation during degradation.^22,26^ Polycaprolactone (PCL), another biodegradable aliphatic polyester that has been used in FDA-approved or FDA-cleared medical devices for specific clinical applications, exhibits higher ductility, slower degradation, and good tissue compatibility.^27,28^ Importantly, polymer blending of PLA with PCL has been reported to buffer acidic degradation products, reduce inflammation, soften the bulk modulus, and prolong degradation time, suggesting a promising strategy to tailor embolic performance by compositional control.^29–33^

In this study, we develop PLA/PCL blend microspheres designed for degradable transarterial embolization. Using an emulsion-based fabrication approach, we prepare blends across multiple PLA/PCL ratios and systematically evaluate their structural, mechanical, and biological properties relevant to endovascular delivery. We investigate how PCL content modulates the microsphere surface morphology and calibratability, quantify mechanical softening through rheological and Young’s modulus measurements, and assess safety via hemolysis and cytotoxicity assays. Functional embolization performance is further examined through thrombus formation testing and a 3D-printed in vitro vascular simulation platform that models distal embolization under controlled conditions. Our results identify an optimized blend formulation that combines nonhemolytic behavior, suggesting enhanced thrombus anchoring potential, stable and extended degradation, and enhanced distal penetration and lodging behavior in the in vitro vascular model. These findings support P­(LA/CL) blend microspheres as a feasible and clinically relevant biodegradable embolic platform, offering a tunable balance between mechanical compliance, biological response, and therapeutic occlusion duration for TAE applications.

## 2 Materials and Methods

### 2.1 Materials

Poly­(d, l-lactide) (PDLLA, hereafter referred to as PLA) was obtained from Corbion (the Netherlands) (GMP grade) with an intrinsic viscosity (IV) of 0.5 dL/g. Poly­(ε-caprolactone) (PCL) was purchased from Corbion (the Netherlands) (GMP grade) with an intrinsic viscosity in the range of 0.35–0.43 dL/g. According to the manufacturer’s specifications, intrinsic viscosity values correspond to medical-grade polymers suitable for biodegradable drug delivery systems. Poly­(vinyl alcohol) (PVA) (Sigma-Aldrich, USA) had a molecular weight of 6000 g/mol and a degree of hydrolysis of 80 mol %. All materials were used as received.

### 2.2 Preparation of P­(LA/CL) Microspheres

P­(LA/CL) microspheres were prepared by a water-in-oil-in-water (W/O/W) double emulsion solvent evaporation method followed by freeze-drying. PLA and PCL were blended at different weight ratios, corresponding to 0, 10, 20, and 30 wt % PCL relative to the total polymer. The formulations were as follows: PLA (1.5 g of PLA, 0 g of PCL), PCL (0 g of PLA, 1.5 g of PCL), P­(LA/CL)­91 (1.35 g of PLA, 0.15 g of PCL), P­(LA/CL)­82 (1.20 g of PLA, 0.30 g of PCL), and P­(LA/CL)­73 (1.05 g of PLA, 0.45 g of PCL). For all groups, 15 mL of dichloromethane (CH_2_Cl_2_) was used as the organic solvent, and a 5 wt % poly­(vinyl alcohol) (PVA) solution was used as the aqueous phase. The PVA powder was dissolved in deionized water under continuous stirring in a water bath to obtain a 5 wt % PVA solution. Separately, the weighed amounts of PLA and/or PCL for each formulation were added into glass vials, and 15 mL of CH_2_Cl_2_ was added. The mixtures were vortexed for 1 min (min) until complete dissolution to form the organic phase.

To generate the primary emulsion, 0.5 mL of 5 wt % PVA solution was added to the polymer–solvent phase and emulsified using an ultrasonic homogenizer at 20 Hz with cycles of 30 s sonication and 3 s pause for a total of 5 min. This process produced a water-in-oil (W/O) emulsion, in which fine aqueous droplets were dispersed within the organic phase containing dissolved P­(LA/CL) in dichloromethane. The resulting primary emulsion was immediately transferred into 150 mL of 5 wt % PVA solution under stirring to form a water-in-oil-in-water (W/O/W) double emulsion, which subsequently led to microsphere formation during solvent evaporation. The secondary emulsion was stirred at 25 °C and 350 rpm for 3 h (h) to allow solvent evaporation and microsphere formation. Afterward, the suspension was distributed into three 50 mL centrifuge tubes. The samples were centrifuged at 3000 rpm for 5 min at 4 °C, the supernatant was discarded, and the pellet was resuspended in deionized water to a volume of 50 mL. This washing step was repeated three times under the same centrifugation conditions to remove residual PVA and solvent. Following the final wash, the supernatant was removed and the microsphere pellets were stored at −80 °C overnight. The frozen samples were then freeze-dried in a vacuum freeze-dryer at −60 °C and 0.02 Torr for 24 h to obtain the final dry microsphere powders.

### 2.3 SEM Analysis

The surface morphology and particle size of PLA, PCL, and P­(LA/CL) microspheres were examined using a scanning electron microscope (SEM). Microsphere powders (10 mg) were placed onto an SEM stub covered with carbon tape, and excess microspheres were removed with an air blower. Samples were gold-coated for 30 s in a sputter coater. Coating was initiated only after the chamber pressure was reduced below 10 mbar, and the system was allowed to return below 10 mbar again after coating before shutting down to avoid coating failure. The gold-coated stubs were then placed into the SEM chamber, which was evacuated to high vacuum before switching on the electron beam. Particle size analysis was performed using ImageJ (version 1.54). For each sample, 300 microspheres were measured, with approximately 100 particles per 40× image from three images, and the average particle diameter and standard deviation were calculated.

### 2.4 FTIR Analysis

Fourier transform infrared spectroscopy (FTIR) was used to analyze the chemical structures of the PLA, PCL, and P­(LA/CL) microspheres. Measurements were carried out using a Nicolet iS5 FTIR spectrometer (Thermo Fisher Scientific). Approximately 100 mg of each sample was ground into a fine powder using an agate mortar and pestle, and pellets were formed by using a tablet press when needed. The attenuated total reflectance (ATR) mode was used for measurements.

### 2.5 Mechanical Testing

The apparent Young’s modulus of PLA, PCL, and P­(LA/CL) microspheres was measured using a texture analyzer (TA.XT Plus, Stable Micro Systems) equipped with a 50 N load cell and a 2 mm cylindrical probe. Microsphere powders were immobilized on the sample platform using double-sided adhesive tape. For each measurement, 50 mg of microspheres were evenly distributed on the tape to form a thin packed particle layer, and excess particles were removed with gentle airflow. This preparation resulted in a loosely packed microsphere layer rather than a strictly isolated monolayer of individual particles. Uniaxial compression was performed at a constant speed (0.1 mm/s) to a target strain of 30% with a 20 s hold at maximum strain. Stress–strain curves were recorded, and Young’s modulus was determined from the slope of the linear elastic region. Because compression was performed on a packed microsphere layer rather than isolated single particles, the calculated Young’s modulus represents an apparent modulus of the microsphere assembly, reflecting both particle elasticity and interparticle interactions. Each formulation was tested in triplicate using fresh microspheres for each run.

### 2.6 In Vitro Degradation and pH Measurement

In vitro degradation was performed to assess mass loss and pH variation associated with PLA-based microsphere degradation. Microspheres were suspended in phosphate-buffered saline (PBS, 20 mg/mL) and incubated at 37 °C. Preweighed centrifuge tubes were used for each condition. At scheduled time points, samples were collected, centrifuged at 3000 rpm for 20 min, and the supernatant was separated for the pH measurement.

The recovered microspheres were washed with 10 mL of deionized water and centrifuged at 3000 rpm for 20 min; this wash cycle was repeated twice. After washing, samples were freeze-dried and weighed to determine remaining mass. Morphological changes over degradation time were examined by SEM. The particle size after degradation was quantified using ImageJ by counting 300 microspheres from three independent 40× images.

### 2.7 Hemolysis Test

Hemocompatibility was evaluated using a hemolysis assay with porcine whole blood. All experiments were performed in triplicate using microspheres from the same preparation batch for each formulation. PLA, PCL, and P­(LA/CL) microspheres were suspended in 0.9% normal saline at a concentration of 10 mg/mL and preincubated at 37 °C for 1 h.

Fresh whole blood was diluted with normal saline prior to testing. Positive and negative controls were prepared by adding 20 μL of diluted blood to 1 mL of distilled water and 1 mL of normal saline, respectively.

After preincubation, 20 μL of diluted blood was added to 1 mL of each microsphere suspension and incubated at 37 °C for 1 h. The mixtures were then centrifuged at 4500 rpm for 10 min to pellet intact erythrocytes. The supernatants were collected, and hemoglobin release was quantified by measuring the absorbance at 545 nm using a microplate reader. The hemolysis ratio (HR%) was calculated according to eq 1:

1 $$HR\%=\left[\left(A_{sample}-A_{nc}\right)/A_{pc}-A_{nc}\right]\times 100\%$$

where *A*_sample_ is the absorbance of the sample, *A*_nc_ is the absorbance of the negative control, and *A*_pc_ is the absorbance of the positive control. Samples with HR% between 0 and 2% were considered nonhemolytic, between 2 and 5% slightly hemolytic, and above 5% hemolytic.

### 2.8 Cytotoxicity Test

Cytotoxicity of PLA and P­(LA/CL) microspheres was assessed by an MTT (3-(4,5-dimethylthiazol-2-yl)-2,5-diphenyltetrazolium bromide) assay. All tests were performed in triplicate using extracts from the same batch. Microspheres were sterilized by UV irradiation for 20 min and extracted in a complete culture medium (10% fetal bovine serum) at 37 °C for 24 h at a material-to-medium ratio of 0.2 g/mL. L929 mouse fibroblasts were seeded in 96-well plates at 1 × 10^4^ cells/well (100 μL) and allowed to attach for 24 h. The medium was removed, cells were washed twice with PBS, and 100 μL of undiluted extract was added to each well; fresh medium served as the control. Cells were incubated with extracts for 24 and 72 h. After incubation, the extract was removed, cells were washed twice with PBS, and 50 μL of MTT solution (1 mg/mL in PBS) was added to each well and incubated at 37 °C for 2 h. The MTT solution was discarded, cells were washed twice with PBS, and 100 μL of DMSO was added to dissolve the formazan crystals. Plates were shaken for 15 min, and absorbance was measured at 570 nm using a microplate reader. Cell viability was calculated relative to the control using the following eq 2:

2 $$cell\;viability\;\left(\%\right)=\left({absorbance}_{sample}/{absorbance}_{control}\right)\times 100\%$$

### 2.9 Thrombus Formation Test

Thrombus-forming capacity was evaluated, because the microspheres are intended for direct blood contact and must remain stably lodged at the target site during embolization. In situ thrombus formation around microspheres can enhance anchoring within vessels and reduce reflux-related nontarget embolization; therefore, the coagulation ability is a critical performance parameter for degradable embolic materials. All experiments were performed in triplicate by using fresh microspheres for each run.

PLA, PCL, and P­(LA/CL) microspheres were preincubated in 0.9% normal saline (10 mg/mL) at 37 °C for 1 h. Fresh whole blood (0.1 mL) and 0.1 M CaCl_2_ (0.1 mL) were then added to initiate coagulation, and samples were incubated at 37 °C for an additional 1 h. Coagulation was terminated by adding 0.5 mL of deionized water and standing for 5 min.

The contents were transferred to a culture dish and nonclotted blood was removed. Thrombi were fixed with 4% formaldehyde (0.5 mL) for 5 min, rinsed repeatedly with deionized water, and dried at 50 °C for 24 h. The dried thrombi were weighed using an analytical balance.

### 2.10 In Vitro Embolization Platform

An in vitro embolization platform was fabricated by 3D printing a photopolymerizable PMMA-based resin. The device comprised a wedge-shaped rectangular channel (length 7 cm) with a linear taper in pore diameter from 300 μm at the inlet to 25 μm at the distal end, modeling progressive arterial branching. Microspheres were suspended in 0.9% normal saline (10 mg/mL). After sealing and saline preflushing, 4 mL of suspension was introduced through the inlet as four sequential 1 mL injections. The platform was then maintained vertically for 30 s to allow particle settling and lodging. The embolization position was defined as the distance from the inlet (0 cm) to the most distal region of dense microsphere accumulation. The corresponding pore diameter at this location was estimated by linear interpolation along the channel taper (300 μm at 0 cm to 25 μm at 7 cm). A schematic representation of the vascular-mimicking model is shown in Figure 6.

### 2.11 Statistical Analysis

Statistical analysis was performed using JASP software (version 0.19). All experiments were conducted at least in triplicate, and data are presented as mean ± standard deviation. One-way analysis of variance (ANOVA) was used to compare differences among groups. Post hoc tests were performed where appropriate, and assumptions such as homogeneity of variances were checked. Significance levels were expressed using asterisks: *p* < 0.05 was denoted by (*), *p* < 0.01 by (**), and *p* < 0.001 by (***). Differences with *p* ≥ 0.05 were considered not statistically significant.

## 3 Results and Discussion

SEM analysis was used to examine the particle size and surface morphology of PLA, PCL, and the P­(LA/CL) blend microspheres prepared by the W/O/W emulsion solvent evaporation method (Figure 1). PLA microspheres exhibited smooth and uniform surfaces with good dispersion, whereas PCL microspheres showed distinct porous structures. As the PCL content increased in the blends (10, 20, and 30 wt %), surface pores became more pronounced, and the overall surface roughness increased. This morphological change may be attributed to partial phase separation between PLA and PCL, which have been reported to exhibit limited miscibility in polymer blends.^34^ The average particle size of PLA microspheres was 120.94 ± 40.01 μm (coefficients of variation (CV) = 33.1%), while PCL microspheres were slightly larger at 134.10 ± 54.90 μm (CV = 40.9%). Among the blends, P­(LA/CL)­91 averaged 112.88 ± 44.90 μm (CV = 39.8%), P­(LA/CL)­82 averaged 122.62 ± 39.81 μm (CV = 32.5%), and P­(LA/CL)­73 averaged 114.08 ± 44.10 μm (CV = 38.7%). The mean particle diameter of all formulations ranged between approximately 110 and 135 μm, with no significant changes attributable to the PCL blending ratio. In addition, PLA and P­(LA/CL) microspheres appeared less aggregated than PCL, suggesting improved dry-state dispersibility that may reduce clumping-related risks such as microcatheter blockage or unintended proximal occlusion. The calculated coefficients of variation (CV) ranged from 32.5% to 40.9%, reflecting the polydisperse particle populations commonly produced by emulsion solvent evaporation methods. The spherical morphology observed in the SEM images is advantageous for embolization applications because spherical microspheres exhibit smoother flow through microcatheters and more predictable intravascular behavior compared with irregular particles. Uniform spherical particles reduce friction during catheter delivery and minimize the risk of catheter blockage, thereby improving procedural reliability. In addition to the particle shape, microsphere size plays a critical role in determining embolization depth, as embolic particles typically lodge in vessels with diameters comparable to their own size, enabling selective occlusion of specific vascular levels within the arterial tree. In the present study, the PLA, PCL, and P­(LA/CL) blend microspheres exhibited mean particle diameter in the range of approximately 110–135 μm, which are suitable for distal embolization in the vascular-mimicking model. These relationships between the microsphere morphology, particle size, and embolization performance have been widely reported in previous embolotherapy studies.^10,35^ The relatively broad particle size distribution observed in this study may influence embolization behavior by enabling multilevel occlusion, where smaller particles penetrate deeper regions while larger particles occlude proximal vessels. While this may enhance overall embolization efficiency, it may also reduce precision and reproducibility in targeted embolization applications.^10,35^

**Figure 1:**
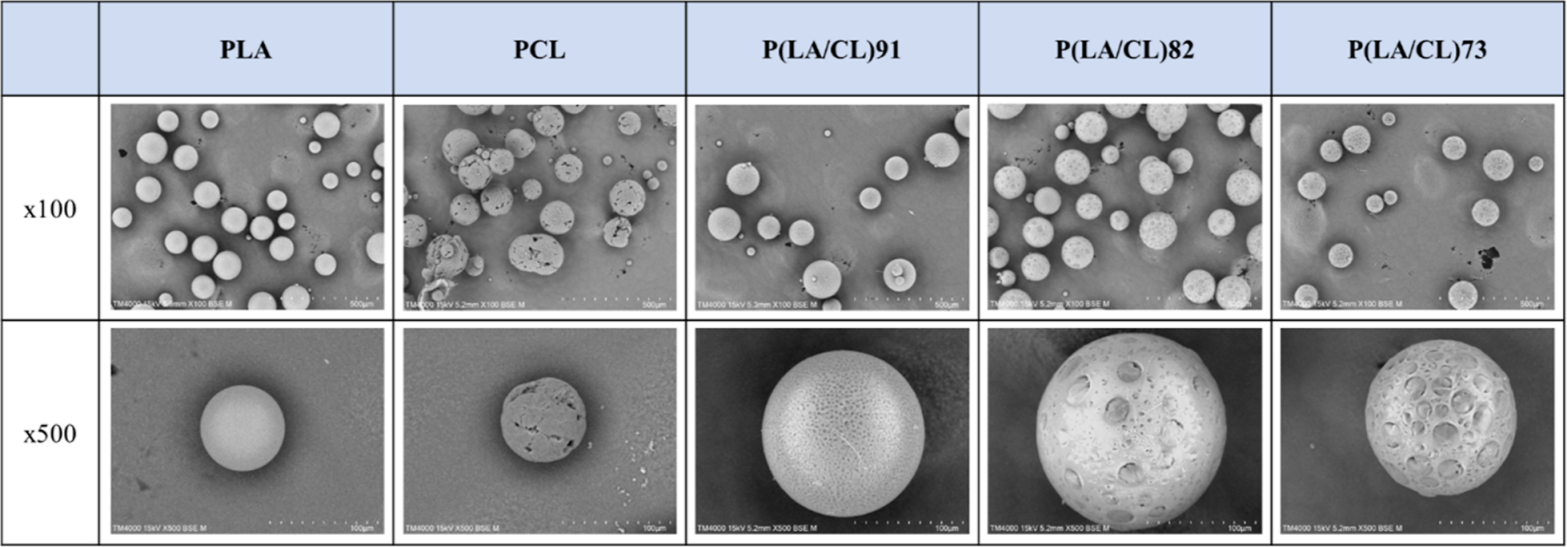
Scanning electron microscopy (SEM) images of PLA, PCL, and P­(LA/CL) blend microspheres (P­(LA/CL)­91, P­(LA/CL)­82, and P­(LA/CL)­73). Top row: low-magnification views (100×) showing overall particle sphericity and size distribution. Bottom row: high-magnification views (500×) highlighting surface morphology.

The surface features observed in the blend microspheres suggest a partial phase separation between PLA and PCL. PLA and PCL are known to exhibit limited thermodynamic miscibility due to differences in their molecular interactions, typically resulting in phase-separated morphologies in which PCL-rich domains are dispersed within PLA-rich matrix. Such a morphology is commonly reported in P­(LA/CL) polymer blends due to the thermodynamic immiscibility between the two polymers.^34^

FTIR analysis was performed to verify the chemical composition of the microspheres and to confirm that the blending process did not introduce chemical reactions between PLA and PCL (Figure 2A). As reported in previous literature, PLA typically exhibits absorption bands corresponding to O–H stretching at 3330–3500 cm^–1^, C–H stretching at 2840–3000 cm^–1^, a strong ester C=O peak near 1715–1730 cm^–1^, C–O–H bending at 1395–1440 cm^–1^, and C–O stretching between 1163–1210 cm^–1^.^36^ PCL, as a structurally similar aliphatic polyester, shows characteristic C–H stretching at 2840–3000 cm^–1^, a C=O ester peak around 1715–1730 cm^–1^, and C–O stretching in the range of 1163–1210 cm^–1^.^37,38^

**Figure 2:**
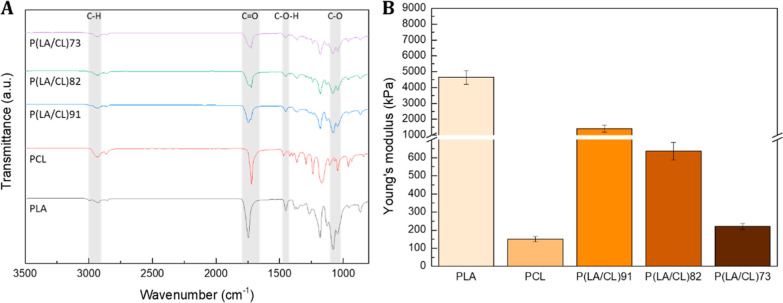
(A) FTIR spectra of PLA, PCL, and P­(LA/CL) blend microspheres P­(LA/CL)­91, P­(LA/CL)­82, and P­(LA/CL)­73. (B) Young’s modulus of PLA, PCL, and P­(LA/CL) blend microspheres measured by uniaxial compression.

The FTIR spectra of PLA, PCL, and the P­(LA/CL) blends revealed that all blend compositions retained the characteristic absorption peaks of both parent polymers. No new peaks or significant peak shifts were observed, indicating that the blending process involved physical mixing rather than a chemical reaction and that the original polymer backbones were preserved. In particular, the C=O and C–O absorption regions in the blends showed overlapping contributions from both PLA and PCL, confirming the coexistence of their functional groups.^29,39^

Compression testing was performed to determine the Young’s modulus of the P­(LA/CL) blends, with PLA and PCL serving as control materials (Figure 2B). PLA exhibited a high Young’s modulus of 4648.53 ± 744.56 kPa, reflecting its inherent stiffness. In contrast, PCL showed a markedly lower modulus of 150.83 ± 26.29 kPa, consistent with its known flexibility.^29^ Among the blend formulations, P­(LA/CL)­73 displayed the lowest modulus at 221.97 ± 28.54 kPa. The overall trend across all compositions demonstrated that increasing PCL content resulted in a progressive reduction in Young’s modulus.^40^ Because the compression measurements were conducted on a packed layer of microspheres rather than isolated single particles, the reported values represent an apparent modulus of the microsphere assembly and may include contributions from particle–particle interactions during compression.

The observed morphological changes in the P­(LA/CL) blend microspheres show a clear correlation with their mechanical and functional properties. As the PCL content increased, the development of a more porous and rough surface structure was observed by SEM, which is consistent with partial phase separation between PLA and PCL.^34^ This morphological evolution is associated with a reduction in Young’s modulus, reflecting increased material deformability.^40^ The reduced apparent modulus is consistent with increased microsphere deformability, which likely contributes to the enhanced distal occlusion behavior observed in the in vitro model. In addition, the increased surface roughness and porosity are known to promote protein adsorption and platelet adhesion, which may underlie the increased thrombus formation observed in PCL-rich blends.^51–53^ These results indicate that morphology appears to play a critical role in influencing both the mechanical behavior and biological performance of the microspheres.

In vitro degradation of PLA, PCL, and P­(LA/CL) microspheres over 24 weeks showed a delayed onset of measurable mass loss (Figure 3A). No measurable erosion was detected at week 2, and only minor, nonsignificant mass loss appeared by week 4. These small variations are within experimental uncertainty and do not represent substantial degradation during the early incubation period. This slow early-stage behavior is consistent with hydrolytic degradation of hydrophobic aliphatic polyesters, where water ingress into the polymer matrix is diffusion-limited, and ester bond hydrolysis proceeds gradually.^41^ By week 16, PLA and the PLA-rich blend P­(LA/CL)­91 exhibited pronounced mass loss, indicating a transition to accelerated bulk degradation, whereas PCL and more PCL-rich blends showed gradual, sustained mass loss consistent with slow bulk hydrolytic degradation. Late-stage acceleration in PLA systems is commonly attributed to autocatalytic hydrolysis: carboxylic acid end groups and soluble acidic oligomers generated during earlier chain scission lower the local pH and promote further ester cleavage.^42^ SEM analysis corroborated these trends, with P­(LA/CL)­73 showing fragmentation beginning at week 12, while other groups mainly displayed surface peeling (Figure 4). Particle diameters remained statistically stable throughout, implying that erosion occurred without significant change in overall sphere size (Figure 3C). The slight pH decrease observed for PLA at week 12, absent in PCL and blends, further supports the onset of localized acidic byproduct accumulation preceding rapid PLA-dominated degradation.^42^ The pH results further support this mechanism (Figure 3B). Prior studies show that PLA degradation can create acidic microenvironments, while PCL degrades slowly and does not generate a comparable acidic milieu.^43^ Blending PLA with PCL has been reported to buffer PLA-driven acidification and reduce inflammation relative to neat PLA.^31^ In this study, only PLA exhibited a slight pH drop around week 12, consistent with lactic acid release during PLA hydrolysis; the subsequent return toward neutrality likely reflects diffusion and dilution of acidic byproducts into the surrounding medium.^44^

**Figure 3:**
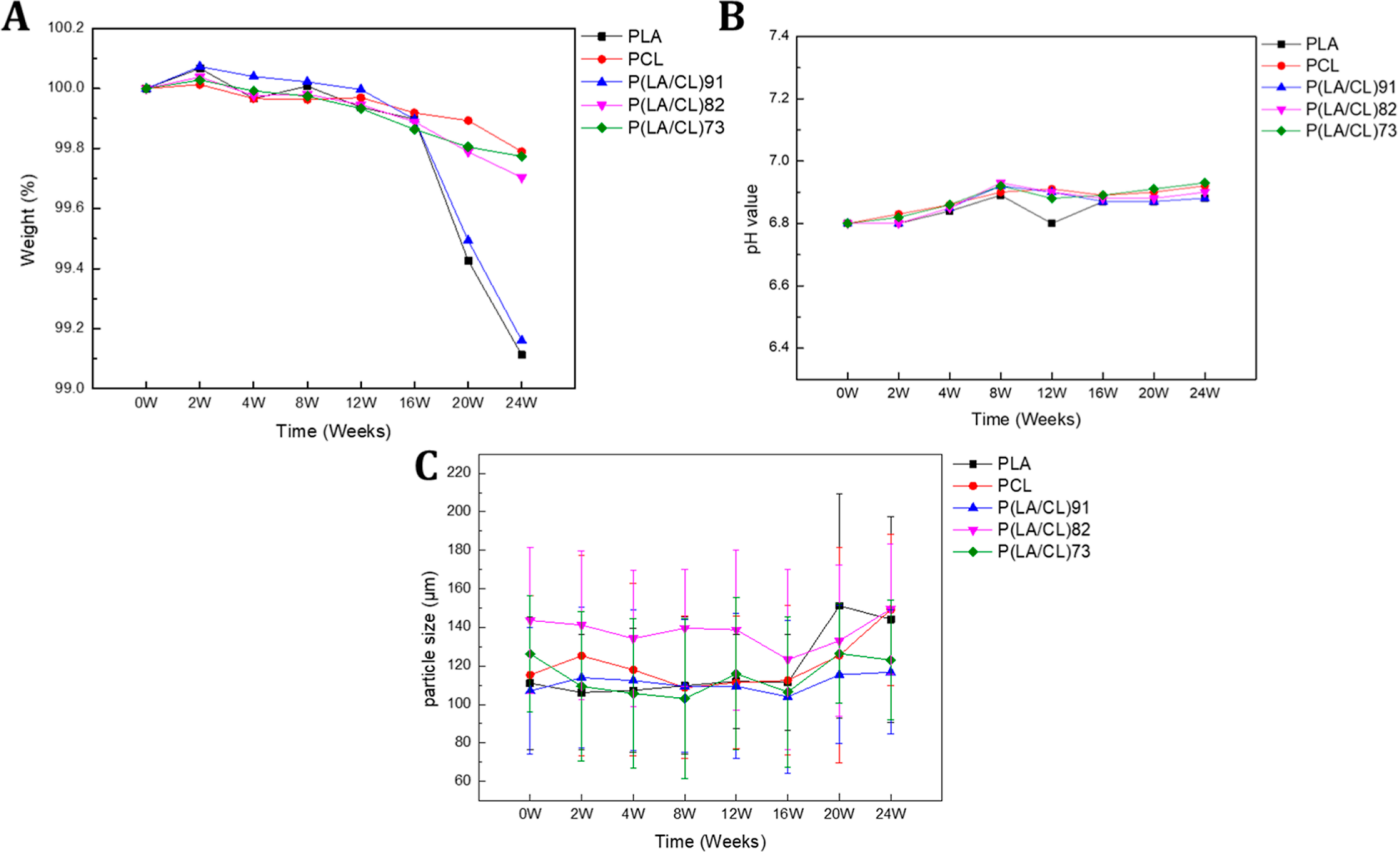
In vitro degradation behavior of PLA, PCL, and P­(LA/CL) blend microspheres (P­(LA/CL)­91, P­(LA/CL)­82, and P­(LA/CL)­73) during 24 weeks of incubation in PBS at 37 °C. (A) Remaining mass (%) as a function of time. (B) pH evolution of the degradation medium over time. (C) Particle diameter during degradation measured from SEM micrographs (*n* = 300 per group).

**Figure 4:**
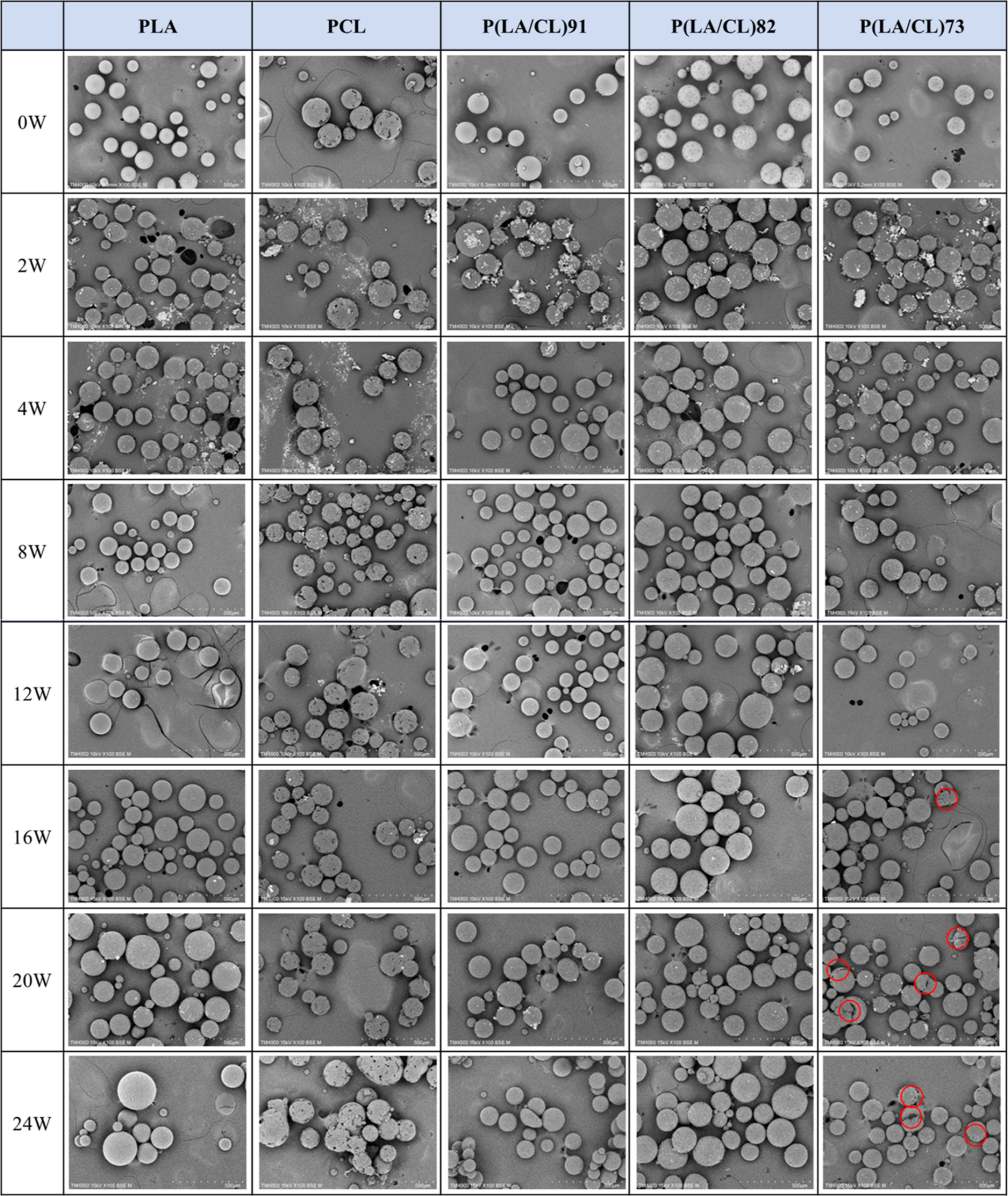
Time-lapse SEM micrographs showing morphological evolution of PLA, PCL, and P­(LA/CL) blend microspheres (P­(LA/CL)­91, P­(LA/CL)­82, and P­(LA/CL)­73) during in vitro degradation in PBS at 37 °C. Images are presented at week 0 (0 W) and after 2, 4, 8, 12, 16, 20, and 24 weeks of incubation.

The degradation behavior observed under PBS-based in vitro conditions is most consistent with bulk hydrolytic degradation rather than with classical surface erosion. In surface erosion, a progressive reduction in particle diameter and continuous surface recession would be expected; however, particle diameters remained statistically stable, and SEM images showed a preserved spherical geometry during early time points, followed by late-stage fragmentation. This pattern indicates internal chain scission preceding measurable mass loss, characteristic of bulk degradation in hydrophobic aliphatic polyesters such as PLA.^45^ Although PLA/PCL blending mitigates PLA-driven acidification during degradation, prolonged occlusion may still induce ischemia-associated inflammation independent of pH changes; therefore, the long-term biological response and optimal occlusion duration must be validated through in vivo studies.

The accelerated mass loss observed for PLA and PLA-rich blends at later time points is consistent with autocatalytic bulk hydrolysis, where the accumulation of acidic chain ends and soluble oligomers promotes further ester cleavage.^46^ While PCL is known to degrade rapidly via enzymatic pathways in vivo,^28^ the present study was conducted in enzyme-free PBS; thus, degradation proceeded primarily via slow nonenzymatic hydrolysis, consistent with the gradual bulk degradation behavior observed.

Hemocompatibility is essential for translating blood-contacting biomaterials, as immediate interaction with blood can trigger coagulation or immune activation; thus, material–blood responses must be carefully evaluated to avoid damage to blood components and loss of function.^44^ The hemolytic behaviors of PLA, PCL, and the P­(LA/CL) blends were evaluated after 1 h of incubation, using distilled water and PBS as positive and negative controls, respectively, and the results are shown in Figure 5A. As expected, the positive control exhibited 100% hemolysis, while the negative control showed 0% hemolysis. All tested materials exhibited hemolysis below 2%, meeting the ASTM F756 criterion for nonhemolytic behavior.^44^ PLA demonstrated the lowest hemolysis rate at 0.17 ± 0.041%, whereas PCL showed a higher value of 1.41 ± 0.002%. The P­(LA/CL) blends exhibited hemolysis rates that increased slightly with higher PCL content, mirroring the trend observed in the pure polymers.

**Figure 5:**
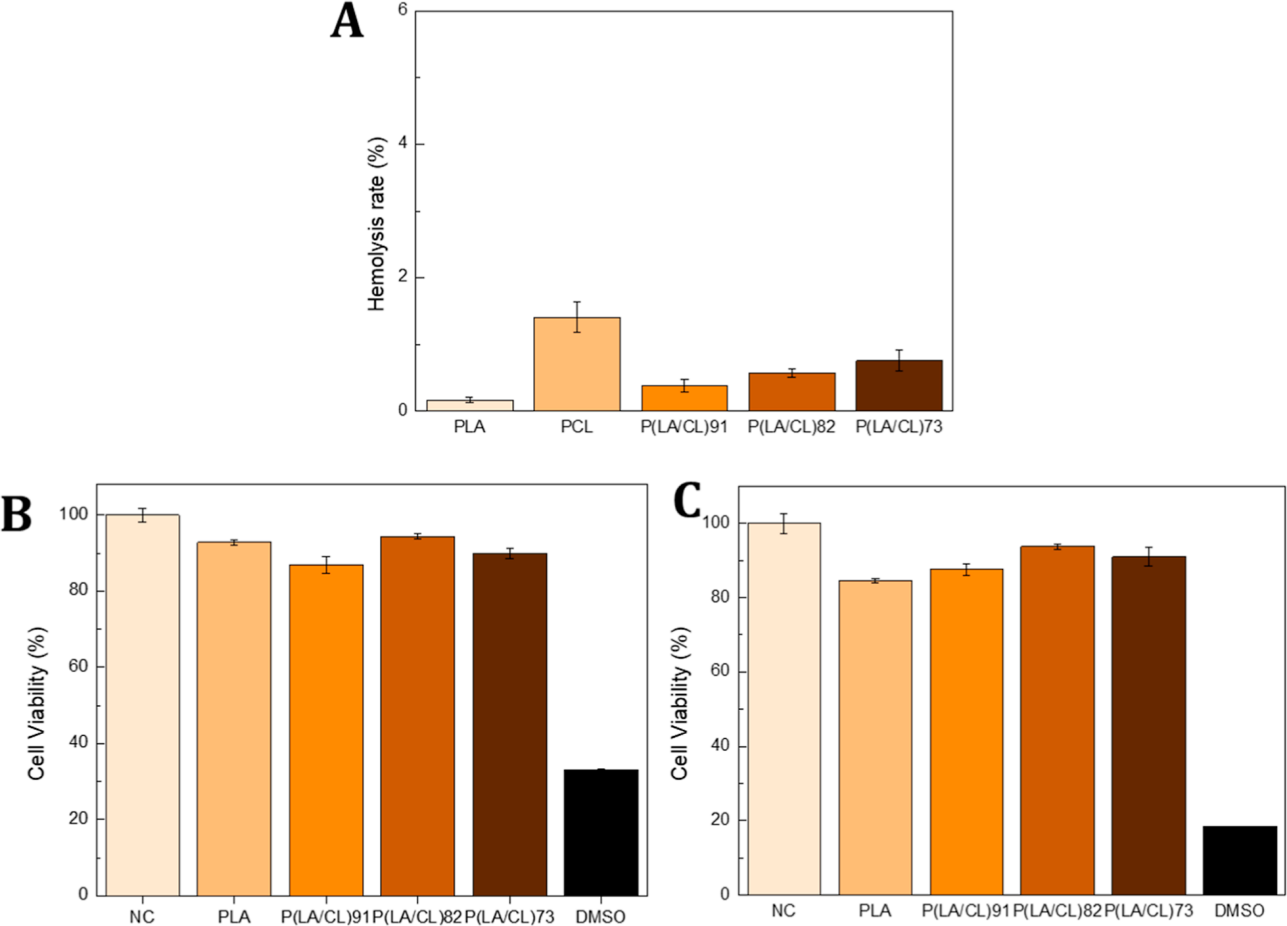
Hemocompatibility and cytocompatibility of neat PLA, neat PCL, and P­(LA/CL) blend microspheres (P­(LA/CL)­91, P­(LA/CL)­82, and P­(LA/CL)­73). (A) Hemolysis ratio after 1 h incubation with porcine blood, showing all formulations below the 2% nonhemolytic threshold. (B) L929 fibroblast viability after 24 h exposure to microsphere extracts. (C) L929 fibroblast viability after 72 h exposure. Data are presented as mean ± standard deviation (*n* = 3). Statistical analysis was performed using one-way ANOVA. No statistically significant differences were observed between groups (*p* ≥ 0.05).

Cytotoxicity testing is a fundamental component of biological evaluation for medical devices, employing in vitro cell culture assays to determine whether a material or its extracts/leachables adversely affect cellular viability, proliferation, and morphology.^47^ Cytotoxicity was assessed following ISO 10993-5 guidelines, which state that materials with cell viability above 70% are considered noncytotoxic.^48^ Extracts of PLA, PCL, and P­(LA/CL) microspheres were incubated with cells for 24 and 72 h, and cell viability was evaluated using the MTT assay. The results presented in Figure 5B,C show that PLA and all P­(LA/CL) blends maintained high levels of cell viability at both time points. No significant cytotoxicity was detected, and none of the samples showed evidence of harmful extractables or residual solvents that could affect the cell metabolic activity.

Thrombogenicity was assessed because embolic microspheres must achieve stable intravascular occlusion through a controlled combination of mechanical lodging and local thrombus formation, which anchors particles and enhances ischemic efficacy while reducing reflux and nontarget embolization risk.^14,49^ Thrombus formation is an important mechanism in embolization therapy because embolic microspheres initially occlude vessels through mechanical lodging, and the subsequent formation of a fibrin-rich thrombus around the particles further stabilizes them within the vascular lumen. This thrombus network anchors the microspheres at the embolization site and reinforces vascular occlusion, thereby improving ischemic efficacy. In addition, stable thrombus formation can reduce particle displacement or reflux during injection, which helps minimize the risk of nontarget embolization. Thrombus formation was evaluated by incubating the materials with whole blood for 1 h and measuring the mass of the resulting blood clots. The results demonstrated clear differences among the groups (Figure 7A). PLA produced a clot weighing 36.33 ± 3.01 mg, whereas PCL generated a larger clot of 45.90 ± 3.50 mg. The P­(LA/CL) blends showed an increasing trend in thrombus mass with rising PCL content. Among the blends, P­(LA/CL)­73 exhibited the highest clot mass, reaching 50.43 ± 12.19 mg, indicating the strongest tendency for thrombus formation. Platelet adhesion on embolic microspheres is promoted by surface charge, wettability, and topography. Platelets are overall electronegative due to sialic acid residues on their membrane glycoproteins.^50^ Platelet attachment to biomaterials is strongly governed by adsorbed plasma proteins (especially fibrinogen), whose conformation and retention depend on surface chemistry and hydrophobicity.^51^ Adhesion initiates faster and more strongly on hydrophobic than hydrophilic interfaces.^52^ The surface microstructure further amplifies thrombosis, as micro/rough features increase the probability of platelet adhesion and thrombus formation.^53^ In this study, PCL microspheres exhibited a greater surface porosity than PLA by SEM, and both polymers are hydrophobic aliphatic polyesters (PLA WCA typically 70–80°, with P­(LA/CL) blend remaining in a hydrophobic regime).^54^ Increasing PCL, therefore, increased a rough/porous contact area on an already hydrophobic surface, providing more sites for protein-mediated platelet attachment, which may contribute to the enhanced thrombus formation observed in PCL-rich blends. From a clinical perspective, moderate thrombus formation is generally desirable for embolization materials because it improves particle anchoring and stabilizes vascular occlusion, while excessive thrombogenicity could increase the risk of unwanted occlusion. In the present study, P­(LA/CL) microspheres, particularly P­(LA/CL)­73, showed a higher clot mass than PLA, suggesting enhanced anchoring potential; however, this in vitro clot-mass assay does not directly define a “clinically acceptable limit.” Therefore, clinical acceptability and thrombogenic safety must ultimately be confirmed in vivo under physiologic flow conditions.

An in vitro, 3D-printed vessel-mimicking platform was used to rapidly and reproducibly assess microsphere lodging and occlusion behavior under controlled conditions, an approach commonly employed to simulate embolization while reducing reliance on animal models.^55–57^ Figure 6 shows a schematic representation of the 3D-printed vascular-mimicking model, illustrating the tapered channel design. The embolization behavior of PLA and the P­(LA/CL) blends was examined using an in vitro vascular simulation platform. The minimum pore size at which each material was able to achieve occlusion is shown in Figure 7B. PLA microspheres caused occlusion at an average pore size of 152.7 ± 2.4 μm, reflecting limited deformability and poorer distal navigation. In contrast, the P­(LA/CL) blends demonstrated progressively improved distal occlusion behavior. P­(LA/CL)­91 occluded pores of approximately 74.7 ± 4.6 μm, while P­(LA/CL)­82 showed occlusion at 63.0 ± 4.5 μm. The best performance was observed in P­(LA/CL)­73, which successfully occluded pores as small as 32.8 ± 4.0 μm, indicating superior deformability and enhanced distal penetration. P­(LA/CL) blends exhibited greater distal embolization than neat PLA. This improvement is likely associated with their lower apparent modulus, although the contribution of particle size distribution and interparticle interactions cannot be excluded. Elasticity (Young’s modulus) is recognized as a principal mechanical determinant of embolic microsphere targeting, and highly deformable microspheres are reported to achieve smoother delivery and more effective distal embolization in vitro and in vivo.^12,58,59^ Although the 3D-printed vascular model enables controlled comparison of embolization behavior, it does not fully replicate physiological conditions such as pulsatile blood flow, blood viscosity, vessel compliance, or immune responses.

**Figure 6:**
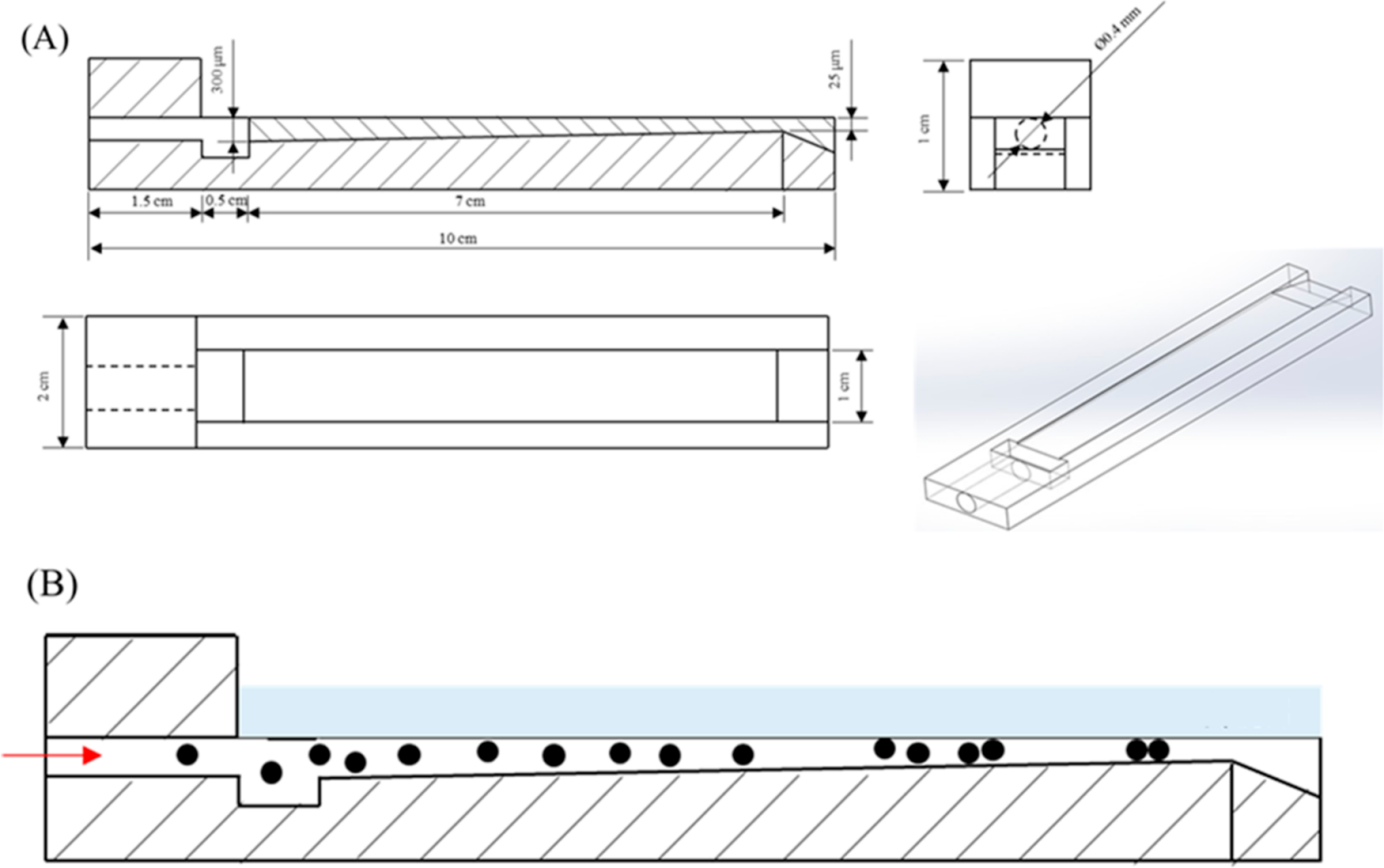
Schematic illustration of the 3D-printed vascular-mimicking model. (A) Geometry of the tapered channel, consisting of a wedge-shaped rectangular structure with a total length of 7 cm and a linear reduction in pore diameter from 300 μm at the inlet to 25 μm at the distal end, designed to simulate progressive arterial branching. (B) Three-dimensional view of the fabricated model, showing the inlet region and tapered channel configuration.

**Figure 7:**
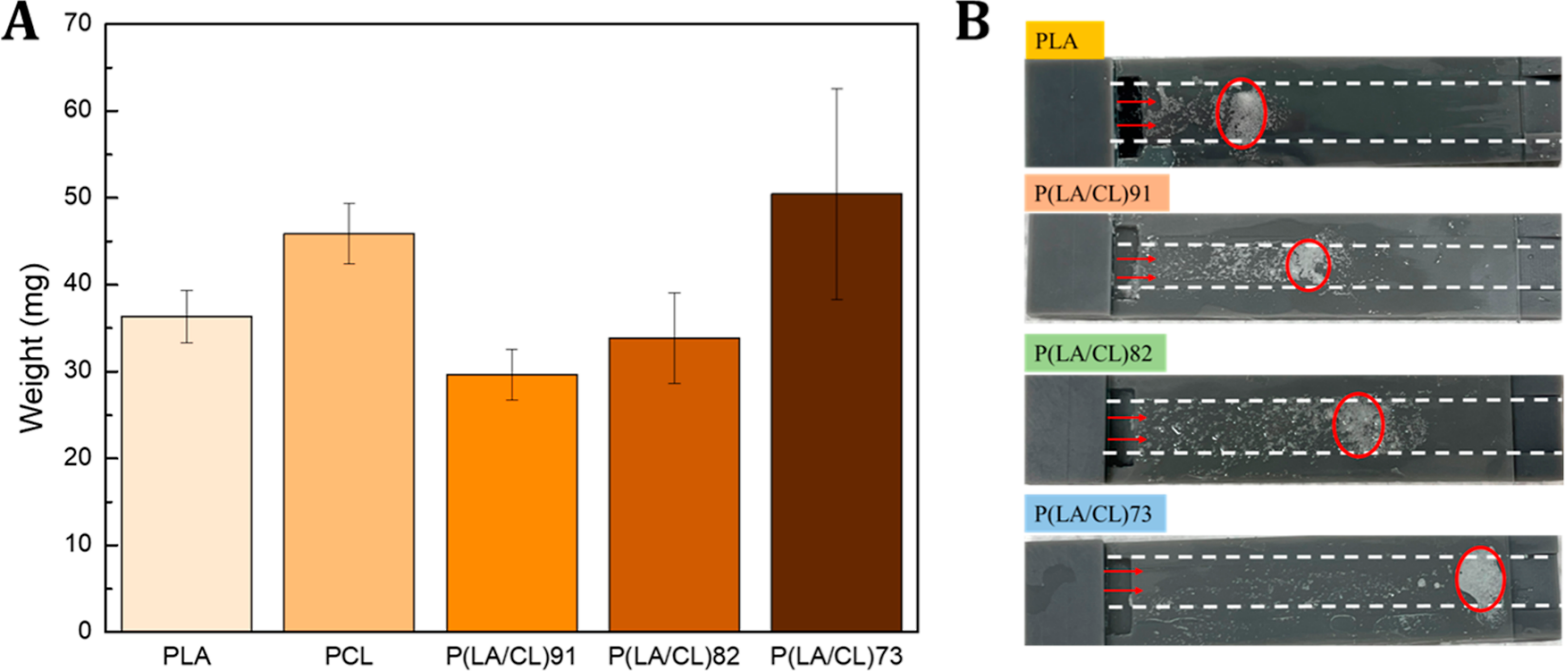
(A) Quantitative assessment of thrombus formation after 1 h incubation of PLA, PCL, and P­(LA/CL) blend microspheres with whole blood showed that P­(LA/CL)­73 produced the largest clots, suggesting a stronger tendency toward thrombus formation. (B) In vitro embolization. Representative 3D-printed tapered-channel images and corresponding embolization distance analysis, where distal dense microsphere accumulation (red circles) defines embolization depth. Data are presented as mean ± standard deviation (*n* = 3). Statistical analysis was performed using one-way ANOVA. No statistically significant differences were observed between groups (*p* ≥ 0.05).

To further contextualize the performance of the developed P­(LA/CL) microspheres, a comparison to representative embolic microspheres reported in the literature is summarized in Table 1.

**Table 1:** Comparison of the Developed Biodegradable P­(LA/CL) Microspheres with Representative Commercially Available and Experimental Embolic Agents Reported in the Literature, Highlighting Differences in the Particle Size, Degradability, Mechanical Properties, Degradation Profile, Embolization Behavior, Advantages, and Limitations

embolic material	particle size	degradability	mechanical behavior	degradation/resorption time	key advantages	embolization behavior	limitations
PVA particles^16^	45–1200 μm	nondegradable	irregular, relatively rigid polymer particles with limited deformability	permanent	durable embolization and long clinical history	proximal embolization	particle aggregation and catheter clogging risk
tris-acryl gelatin microspheres (embosphere)^10,35^	40–1200 μm	nondegradable	elastic hydrogel beads with controlled deformability	permanent	calibrated size distribution	predictable distal embolization	nonresorbable
gelatin sponge particles (gelfoam)^18^	irregular fragments	biodegradable	soft porous material	3–6 weeks	temporary embolization widely used clinically	proximal temporary occlusion	rapid degradation may cause recanalization
degradable starch microspheres (DSM)^17,60^	∼50 μm	biodegradable	soft particles	30–60 min	very rapid temporary embolization	distal microvascular temporary embolization	extremely short embolization window
PLGA microspheres^19,61–63^	tunable (micrometer-scale depending on the fabrication method)	biodegradable	moderate stiffness depending on the polymer composition	weeks–months	tunable degradation and drug-loading capability	size-dependent embolization; biodegradable temporary occlusion	acidic degradation products may cause inflammation
P(LA/CL) microspheres (this study)	110–135 μm	biodegradable	tunable modulus	sustained degradation observed up to 24 weeks	tunable mechanical compliance	enhanced distal penetration in the in vitro model	drug loading was not investigated

Conventional embolic agents such as poly­(vinyl alcohol) (PVA) particles and tris-acryl gelatin microspheres (embosphere) are nondegradable and provide permanent vascular occlusion. In contrast, temporary embolic agents such as gelatin sponge particles (gelfoam) and degradable starch microspheres (DSMs) exhibit rapid degradation and shorter embolization duration. Biodegradable polymer microspheres such as PLGA have also been explored due to their tunable degradation behavior. In comparison, the P­(LA/CL) microspheres developed in this study combine biodegradable characteristics with a tunable mechanical compliance and controlled degradation behavior while also demonstrating enhanced distal penetration in the in vitro model.

Recent studies indicate that embolic microsphere development is evolving toward systems with improved deformability and a controlled biodegradation. A study on highly elastic embolic microspheres demonstrated that increased deformability can enhance embolization behavior, highlighting the importance of mechanical compliance.^64^ In addition, recent reports on advanced microspheres and hydrogel platforms for interventional therapy highlight the integration of controlled degradation, therapeutic functionality, and improved interaction with the tumor microenvironment.^65^ In this context, the PLA/PCL system developed in the present study can be considered a biodegradable and mechanically tunable base platform that aligns with these emerging directions and may be further extended to advanced embolic applications.

The present study has several limitations that should be acknowledged. First, all evaluations were conducted under in vitro conditions, which do not fully replicate the complex physiological environment of the vascular system, including pulsatile blood flow, shear stress, vascular compliance, and immune responses. Second, the in vitro embolization platform represents a simplified model and does not account for blood rheology, catheter-based delivery, endothelial interactions, or coagulation dynamics under flow conditions. Therefore, the observed embolization behavior should be interpreted as a comparative assessment of particle lodging in a controlled geometry rather than a direct predictor of in vivo performance. Third, the microspheres exhibited a relatively broad particle size distribution, which may influence embolization precision and reproducibility, and may confound the independent contribution of mechanical properties to distal penetration. Fourth, although degradation behavior and bulk pH changes were evaluated, the study did not directly assess local inflammatory responses, macrophage activation, protein adsorption, or platelet activation. In addition, bulk pH measurements may not fully reflect the localized microenvironment at the material–tissue interface. Finally, while sustained degradation and prolonged vessel occlusion may enhance embolization durability, excessively long occlusion may also increase the risk of prolonged ischemia, tissue necrosis, and associated inflammatory responses. Therefore, the optimal degradation profile of embolic microspheres should be tailored to specific clinical indications, balancing effective therapeutic occlusion with timely recanalization to minimize adverse effects.

Further validation under physiologically relevant conditions is required to assess the long-term inflammatory response, vascular remodeling, and recanalization behavior associated with the developed microspheres. The development of advanced in vitro platforms incorporating pulsatile flow, blood-mimicking fluids with appropriate rheological properties, and compliant vessel-like materials would improve the predictive capability of the embolization performance. Improving particle size uniformity through controlled fabrication techniques, such as microfluidics, may enable a more precise evaluation of size-dependent and mechanical effects while enhancing embolization precision and reproducibility. Surface modification strategies, including functionalization with bioactive or hemostatic agents (e.g., thrombin, fibrin, or collagen), could further enhance protein interactions, thrombus formation, and the embolization efficiency. In addition, the PLA/PCL platform offers the potential for integration with drug delivery systems. The incorporation of therapeutic agents, radiopaque materials, or stimuli-responsive components may enable the development of multifunctional embolic microspheres for chemoembolization and advanced embolization applications. The tunable mechanical and degradation properties of the PLA/PCL system provide a versatile foundation for the development of next-generation embolic materials with an enhanced therapeutic functionality.

## 4 Conclusion

In this study, P­(LA/CL) blend microspheres were fabricated at controlled ratios and evaluated as biodegradable embolic agents for TAE. Increasing PCL content increased surface porosity and progressively reduced Young’s modulus without altering the microsphere size range, yielding more compliant particles for distal delivery. All blends were nonhemolytic (HR < 2%) and cytocompatible, while thrombus formation rose with PCL fraction; P­(LA/CL)­73 generated the largest clots and showed the deepest distal embolization. Degradation proceeded slowly over 24 weeks with minimal pH change, supporting sustained occlusion without significant acidification. These results identify P­(LA/CL)­73 as the most balanced formulation and a strong candidate for next-generation degradable embolization.
